# Genetic and Epigenetic Biomarkers of Immune Checkpoint Blockade Response

**DOI:** 10.3390/jcm9010286

**Published:** 2020-01-20

**Authors:** Qingyang Xiao, André Nobre, Pilar Piñeiro, Miguel-Ángel Berciano-Guerrero, Emilio Alba, Manuel Cobo, Volker M. Lauschke, Isabel Barragán

**Affiliations:** 1 Group of Pharmacoepigenetics, Department of Physiology and Pharmacology, Karolinska Institutet, 171 77 Stockholm, Sweden; xiao.qingyang@ki.se (Q.X.); andrenobre@sapo.pt (A.N.); 2 Section of Immuno-Oncology, Medical Oncology Service, University Hospitals Regional and Virgen de la Victoria, Biomedical Research Institute of Malaga (IBIMA), 29010 Málaga, Spain; itspilipineiro@gmail.com (P.P.); migueberci@gmail.com (M.-Á.B.-G.); emilioalbac@gmail.com (E.A.); manuelcobodols@yahoo.es (M.C.); 3 Group of Personalized Medicine and Drug Development, Department of Physiology and Pharmacology, Karolinska Institutet, 171 77 Stockholm, Sweden; volker.lauschke@ki.se

**Keywords:** immunotherapy, predictor, resistance, epigenetics, stroma, melanoma, non-small-cell lung cancer

## Abstract

Checkpoint inhibitor therapy constitutes a promising cancer treatment strategy that targets the immune checkpoints to re-activate silenced T cell cytotoxicity. In recent pivotal trials, immune checkpoint blockade (ICB) demonstrated durable responses and acceptable toxicity, resulting in the regulatory approval of 8 checkpoint inhibitors to date for 15 cancer indications. However, up to ~85% of patients present with innate or acquired resistance to ICB, limiting its clinical utility. Current response biomarker candidates, including DNA mutation and neoantigen load, immune profiles, as well as programmed death-ligand 1 (PD-L1) expression, are only weak predictors of ICB response. Thus, identification of novel, more predictive biomarkers that could identify patients who would benefit from ICB constitutes one of the most important areas of immunotherapy research. Aberrant DNA methylation (5mC) and hydroxymethylation (5hmC) were discovered in multiple cancers, and dynamic changes of the epigenomic landscape have been identified during T cell differentiation and activation. While their role in cancer immunosuppression remains to be elucidated, recent evidence suggests that 5mC and 5hmC may serve as prognostic and predictive biomarkers of ICB-sensitive cancers. In this review, we describe the role of epigenetic phenomena in tumor immunoediting and other immune evasion related processes, provide a comprehensive update of the current status of ICB-response biomarkers, and highlight promising epigenomic biomarker candidates.

## 1. Introduction

Immunotherapy constitutes a major breakthrough in cancer treatment. In particular, approaches based on inhibiting programed death-1 (PD-1)/PD-L1 and cytotoxic T-lymphocyte-associated protein 4 (CTLA4) are the most rapidly growing drug class [1]. In recent years, the reprogramming of immune checkpoint receptor (ICR) expression on the surface of T cells emerged as a critical mechanism of tumor cell immune-evasion [2]. ICRs are a class of co-stimulators (CD27, CD28, and CD137) and co-inhibitory receptors (e.g., PD-1, CTLA-4, lymphocyte activation gene 3 (LAG-3)) that regulate T cell response quality [3]. The most widely used immune checkpoint blockade (ICB) therapeutic strategies target the PD-1/PD-L1 and CTLA4 axes to regulate anti-tumor immune activity with demonstrated clinical benefits [4]. The interaction between PD-1 in the cytotoxic T lymphocyte and PD-L1 in the cancer cell decreases the activity of T cells by several mechanisms, including the inhibition of the T cell receptor downstream signaling [5,6], the enhancement of regulatory T cells [7], and the decrease in B cells and natural killer activities [8]. Another important ICR is CTLA-4, which impairs T cell activation by outcompeting the co-stimulatory receptor CD28 [9]. PD-1 and CTLA-4 blockade restores the anti-tumor immune response by inducing the expansion of exhausted-like tumor-infiltrating CD8 T cells; in addition, CTLA-4 blockade rescues Th1-like CD4 effector T cells and could be implicated in the enhancement of CD8 infiltration and cytolytic activity as well as the formation of memory T cells [10].

ICB is effective in multiple malignancies with strong immunogenicity including melanoma and non-small-cell lung cancer (NSCLC). Indeed, the U.S. Food and Drug Administration (FDA) and the European Medicines Agency (EMA) have approved the clinical use of ICB drugs for melanoma, NSCLC, renal cell carcinoma, head and neck squamous cell cancer, Hodgkin’s lymphoma, urothelial carcinoma, gastric cancer, cervical cancer, hepatocellular carcinoma, primary mediastinal large-B-cell lymphoma, microsatellite instability-high/deficient mismatch repair cancer, and Merkel cell carcinoma. In 2019, the first-line anti-PD-1 treatment was approved for patients with stage III NSCLC that were not susceptible to surgery, definite chemoradiation, or present metastasis, and that complied with an epidermal growth factor receptor (EGFR) or anaplastic lymphoma kinase (ALK) wild-type phenotype and PD-L1 expression (Table 1). Interestingly, more than 20 clinical trials are currently ongoing for various novel oncological indications (Table 2). Although a considerable fraction of patients demonstrate an objective clinical response, the majority of patients do not respond appropriately to ICB therapy because of primary or acquired treatment resistance [11,12]. The least efficient line of treatment so far was anti-CTLA4, with ~85% of non-responding patients [13,14,15], followed by anti-PD-1 approaches with a response of ~40% [16,17]. Combination regimens are related to better percentages of response (~50%) but also higher toxicity [18,19]. Hence, biomarkers predicting ICB response are imperatively needed.

There is increasing evidence of the important role of epigenetic marks, such as 5mC and 5hmC, in carcinogenesis [20,21,22]. Interestingly, 5mC is also of critical importance for regulating T cell proliferation and maintaining differentiation in cytotoxic and helper T cells [23], whereas 5hmC dynamically changes during T cell differentiation [24]. Recently, a specific role of 5hmC deposition in key immune genes has been reported for the activation and differentiation of T lymphocytes after antigen presentation. Interestingly, the variations in 5hmC were more dynamic than those in 5mC [25]. Importantly, tumor cells such as hepatocellular carcinoma cells show a cancer-specific methylome and hydroxymethylome profile [26], indicating the potential of these epigenetic marks to serve as diagnostic or prognostic biomarkers [27]. Indeed, we have recently shown that specific patterns of DNA methylation, named as the “EPIMMUNE” signature, that can be been further subrogated to a single CpG variant in *FOXP1*, have been associated with clinical benefit in NSCLS patients receiving ICB [28]. Here, we will revisit the current research on and clinical use of ICB biomarkers, and we will critically review 5mC and 5hmC as potential biomarkers of response to cancer immunotherapy.

## 2. Induction of Inhibitory Immune Checkpoints (ICs) as a Major Mechanism of Tumor Immune Evasion

The “immunoediting” hypothesis conceptualizes the evolution of the tumor cells under immune pressures towards expansion and immune surveillance escape of the tumor [29]. Several factors contribute to this immune evasion. The tumor microenvironment (TME) can be immunosuppressive per se, facilitating tumor progression with cytokines, chemokines, and inhibitory factors [30]. For example, VEGFA can upregulate PD-1 expression on CD8+ T cells, while TGF-β enhances PD-L1 expression on tumor cells [31,32]. In addition, immune-cold tumors could prevent effector T cells from entering into the tumor, in which case the patients are mostly irresponsive to cancer immunotherapy. Moreover, the TME can also recruit immunosuppressive immune cells including regulatory T cells, myeloid-derived suppressor cells (MDSCs), and tumor-associated macrophages to evade immune clearance [33,34,35].

Besides, tumor cells could diminish the number of T-cell-recognized neoantigens by reducing their gene expression or losing the mutant allele in a selection process triggered by the pressure of the immune attack [36]. Other mechanisms related to tumor immunoediting include the downregulation of interferon-γ (IFN-γ) or antigen presentation and recruitment pathways [37,38,39].

Finally, one of the major mechanisms of immunosuppression that occurs in the context of tumorigenesis and tumor growth is the upregulation of multiple inhibitory co-receptors (ICRs) that create a series of interactions on the tumor–stroma interface and within the stroma itself, leading to the blockade of the immune attack and exhaustion of the T cells [12]. T cell exhaustion was first observed in mice infected with certain strains of lymphocytic choriomeningitis virus (LCMV) [40]. Strikingly, these viruses escaped elimination by rapidly inducing most of the CD8^+^ effector T cells (T_eff_), thereby resulting in the depletion of this specific antiviral T cell population within a few days and, consequently, persistent infection. The key hallmarks of T cell exhaustion are the expression of ICR, leading to loss of effector functions and failure to transition into the memory T cell pool [41]. 

Importantly, recent research demonstrated that T cell exhaustion is of central importance in various cancers similarly to the exhaustion occurring during chronic infection. In both cases, chronic antigen stimulation triggers co-expression of high levels of multiple inhibitory receptors, including PD-1, CTLA-4, LAG-3, and T-cell immunoglobulin and mucin domain-3 (TIM-3) [42]. The PD-1/PD-L1 signaling axis plays a predominant role in negative regulation of immune response. For instance, when co-expressed with TIM-3, PD-1 decreases the secretion of various pro-inflammatory cytokines, such as IL-2, IFN-γ, and TNF, and results in T cell tolerance towards tumor cells in acute myelogenous leukemia, colon adenocarcinoma, and melanoma [43,44,45]. Another clinically relevant ICR is CTLA4 that, as PD-1, but non-redundantly, establishes immune inhibitory interactions for the blockade of the co-stimulation of T cell activation, and it maintains the peripheral immune tolerance. When CTLA-4 and PD-1 are co-blocked in B16 melanoma cells vaccinated with B16-Flt3-ligand (Fvax), these agents synergistically increase the ratio of T_eff_ to T_reg_ and myeloid-derived suppressor cells, as well as the production of T cells that secrete IFN-γ and TNF-alpha. This triggers an inflammatory cascade that enhances tumor rejection and diminishes tumor-induced immune suppression [46]. Given this prominent role of the PD-1/PD-L1 and CTLA4 pathways in cancer immune evasion, anti-PD-1/PD-L1 and anti-CTLA4 drugs, and their combinations, have become the current paradigm of IBC-based cancer immunotherapy.

## 3. Mechanisms of Clinically Targeted ICR Signaling pathways

### 3.1. PD-1 Signaling

PD-1 is broadly expressed on T-lymphocytes, B-lymphocytes, antigen-presenting cells, NK cells, and macrophages [47,48]. PD-1 is usually deemed as a dominant inhibitory ICR. Unlike CTLA-4, activation of the PD-1 signaling pathway mainly occurs in the effector phase of the adaptive immune suppression cascade, and it blocks the capacity of cytotoxic T cell to eliminate cancer cells [9]. 

Binding of PD-1 to PD-L1 moreover blocks CD28 [49] and T cell receptor (TCR) signaling activation and the contact of T lymphocytes and dendritic cells (DCs) [50]. In tumor-associated macrophages, a high PD-1 expression level causes decreased macrophage phagocytosis [48]. Expression of its ligand PD-L1 on tumor cells leads to T_eff_ cytolysis resistance and reduced transcript levels of granzyme A and perforin [51,52]. Activated PD-1 signaling inhibits conversion of T_eff_ into the memory T cell pool by pro-apoptotic activities of T_eff_ with upregulation of BCL-2-interacting mediator of cell death (BIM) [41]. Furthermore, PD-1 signaling paralyses CD4^+^ and CD8^+^ T cell motility during exhaustion by stabilizing the immunological synapse formation [53]. Besides, PD-1 promotes the suppression of melanoma antigen-specific CTL that is mediated by CD4+CD25Hi regulatory T cells [54]. This relevance of PD-1 signaling in tumor immunity is exemplified by the identification of PD-1 expression in CD8^+^ T cells as a biomarker of resident tumor reactive T cell subpopulations in advanced melanoma and cervical cancer patients [55,56].

### 3.2. CTLA-4 Signaling

In contrast to the broad expression of PD-1, cytotoxic T lymphocyte antigen-4 (CTLA-4) is mainly expressed on T_reg_ cells and controls immunological self-tolerance and T_reg_-induced immunosuppression [57,58]. On one hand, CTLA-4 inhibits CD28-dependent T cell activation and survival, resulting in reduced levels of IL-2, IL-4, TNF-α, and IFN-γ as well as diminished proliferation of CD8^+^ and CD4^+^ T cells [49,59,60]. Also, CTLA-4 interaction with CD80 and CD86 expressed by conventional T cells (Tconv) increases their susceptibility to T_reg_-mediated suppression [61]. Furthermore, CTLA-4 inhibits CD86/80 expression on DC and impairs antigen priming (initial T cell activation) by excluding T_reg_ physical attachment with DCs and conventional T cells (T_convs_) [62]. In addition, CTLA-4^+^ CD4^+^ T cells have shorter-term interactions with DCs under antigen exposure than that of CTLA-4^-^ CD4^+^ T cells, causing a decrease in IL-2 levels and proliferation [63]. Lastly, CTLA-4 restricts follicular helper T cell (T_fh_) differentiation by controlling the level of CD28 engagement [64].

## 4. Molecular Underpinnings of ICB Failure

Although ICB has revolutionized the therapy of cancer, significant fractions of patients are insensitive, or eventually develop resistance to ICB [39]. Overall, 9% of patients receiving anti-PD-1/PD-L1 monotherapy shows hyper-progressive tumor aggression with poor overall survival [65]. Resistance is generally driven by intra-tumor heterogeneity coupled to selection of resistant cells. Generally, intratumor heterogeneity results in molecularly different cancer cell subpopulations, among which a fraction is insensitive to cancer therapy [66]. With sensitive tumor cells being killed, the surviving resistant cells drive tumor progression. In the context of ICB, this tumor heterogeneity can be particularly important, given that many different tumor intrinsic and stromal factors collude in the final clinical outcome. Tumor heterogeneity has been described for key modulators of the ICB response such as PD-L1 [67], and the neoantigens and tumor clonality has already been reported as a predictor of enhanced response to anti-CTLA4 and anti-PD-1 treatment in NSCLC [68].

### Resistance Mechanisms

A crucial tumor-intrinsic factor leading to ICB resistance is the low neoepitope load that generally leads to minimal immune reinvigoration with both CTLA-4 and PD-1/PD-L1 blockade [69,70,71,72]. Interestingly, alterations in the epitope or mutation load during treatment with ICB has been reported as related to response. In the context of NSCLC patients at the moment of response to anti-PD-1 treatment, a reduction of the number of clonal mutations and T cell repertoire evenness is proportional to the response, being 19% the average fraction of remaining variants in those patients with complete and partial response, and 101% the fraction for patients presenting disease progression [73]. Interestingly, the tumor immunoediting induced by anti-PD-1 or anti-PD-1/anti-CTLA-4 therapies has also been related to a loss of dominant mutation-associated neoantigens in initially responding patients that developed acquired resistance, suggesting the further evolution to tumors with diminished immunogenicity [74].

In addition, there are a number of specific genetic and transcriptomic aberrations that have been proposed as candidates for response biomarkers (Table 3). Prototypic oncogenic pathways include amplifications in the MDM2 gene family, alterations in EFGR alterations, which are associated to hyper-progressive disease after anti-CTLA-4 or PD-1/PD-L1 treatment [75], together with alterations that imply the activation of the canonical Wnt/β-catenin signaling pathway [39]. The latter marks a “non-T-cell inflamed” tumor microenvironment. In addition, Wnt/β-catenin signaling could directly inhibit T cell activation [76].

**Figure 1:**
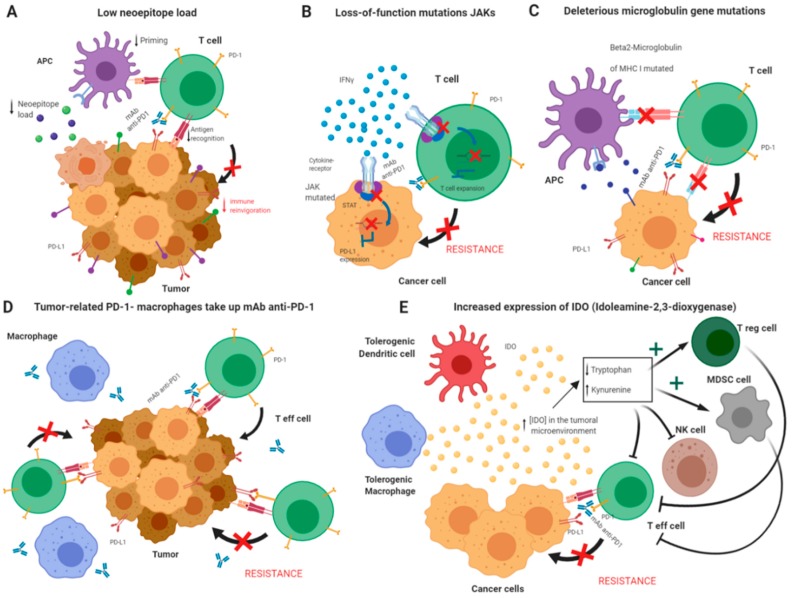
Representative mechanisms of resistance to anti-PD-1 immune checkpoint blockade. (**A**) A low epitope load in the tumor cells normally drives to a minimal immune reinvigoration due to a lower capacity of antigen presenting cells (APC) to present antigen to T cells (low priming) and a lower cytotoxic T cell recognition of the tumor cell antigen. (**B**) Loss-of-function mutations of Janus kinase (JAKs) desensitize the T cells to the IFN-γ exposure and dramatically decrease the expression level of PD-L1 through lack of activation of the transcription factor STAT. This decrease in PD-L1 leads to both primary and acquired resistance of PD-1 blockade therapy, given that the reinvigoration capacity of T cell through reactivation of the PD-1/PD-L1 axis is abrogated. (**C**) Deleterious mutations in the gene encoding β2 microglobulin (an MHC class I subunit) lead to loss of antigen presentation, producing resistance to anti-PD-1 drugs. (**D**) The propensity of the tumor-related PD-1 macrophages to take up anti-PD-1 monoclonal antibodies causes the capture of the anti-PD-1 antibody even from the surface of the PD-1^+^CD8^+^ T cells that already bound the drug. This impedes or reverts the anti-PD-1/PD-1 interaction at the cytotoxic T cell provoking resistance to the treatment. (**E**) In the “escape” phase of the tumor immunoediting, when the tumor is clinically manifested, tolerogenic dendritic cells, myeloid-derived suppressor cells (MDSCs), and tumor-associated macrophages secrete indoleamine-2,3-dioxygenase (IDO), which decreases tryptophan and increases kynurenine. These molecules inhibit effector T cells and NK functions and stimulate regulatory T cells, provoking immunosuppression and enhancing the tolerogenicity of macrophages and dendritic cells. IDO1 also enhances the expansion and activation of MDSCs. All previous alterations suppress the activity of anti-tumor effector T cells.

Among the immune-related mechanisms (Figure 1), loss-of-function mutations of Janus kinase (JAKs) desensitize the T cells to the IFN-γ exposure and dramatically decrease the expression level of PD-L1, which is normally transcribed in response to IFN-γ via Signal Transducer and Activator of Transcription (STAT) activation. This decrease in PD-L1 leads to both primary and acquired resistance of PD-1 blockade therapy, given that the reinvigoration capacity of T cells through reactivation of the PD-1/PD-L1 axis has been abrogated [37,110]. Disruption of the IFN-γ pathway can also occur through a transcriptional dysregulation of several genes used to build the “IFN-γ-associated gene expression score”, which indicates to which extent the tumor microenvironment is “T cell inflamed”. This score is predictive of response to pembrolizumab (anti-PD-1 ab), and the lack of IFN-γ-associated gene expression is associated with lack of clinical benefit in ICB treatment of melanoma, NSCLC, and gastric cancers [39]. This scenario is associated with response to treatment with anti-PD-1 antibodies [90], and such transcriptomic signatures are considered both prognostic and predictive [111]. Indeed, impairment of the IFN-γ pathway through knockdown of Ifgr1 after anti-CTLA4 treatment shows enhanced tumor growth and reduced survival in mice [112]. In addition, deleterious mutations in the gene encoding β2 microglobulin (a MHC class I subunit) have also been described in anti-PD-1-antibody-resistant patient samples and cell lines [37,113].

Another immune-related proposed mechanism of resistance to PD-1 inhibition is the propensity of tumor-related PD-1 macrophages to take up the anti-PD-1 monoclonal antibodies, even those that are already PD-1-engaged on the membrane of the PD-1^+^CD8^+^ T cells [114]; in such a scenario, the co-receptor PD-1 and PD-L1 interaction between the T and tumor cells cannot not be disrupted and the immune blockade not unleashed.

Finally, upregulation of other co-inhibitory ICs is among other possible causes of acquired resistance in PD-1 blockade [115], as well as in CTLA-4 therapy, where the activation of tumor indoleamine-2,3-dioxygenase (IDO) constitutes an important resistance mechanism that results in suppression of T cells and NK cells in the TME, stimulation of regulatory T cells, and enhancement and expansion of myeloid-derived suppressor cells (MDSCs) [116,117]. Indeed, IDO-deficient mice increase CD4^+^ and CD8^+^ effector T cell infiltration in the tumor microenvironment and show better anti-CTLA-4 therapy effects than that of the wild-type [118]. 

## 5. ICB Response Biomarker Candidates

Despite substantial progress in our understanding of immune checkpoints and the development of specific immune checkpoint inhibitors, many patients with immunogenic tumors are insensitive to ICB. In addition to this lack of efficacy, serious adverse effects and high treatment cost incentivize the search for biomarkers that allow a preemptive identification of ICB responders [119]. Combination of static biomarkers obtained in pre-treatment and dynamic biomarkers for monitoring and further clinical stratification are supposed to be incorporated into ICB treatment regimens [120]. Currently, response biomarker candidates for ICB have been discovered on several levels, including genomic, transcriptomic, and proteomic levels, along with immunological parameters [121,122] (Table 3).

### 5.1. Solid Biopsy Biomarker Candidates

Clinical biomarkers that might be useful for the prediction of ICB response have been uncovered at different biological levels (cellular, protein, transcript, gene), in different locations (tumor, peripheral blood), and associated to the biology of both immune and tumor-related cell populations.

#### 5.1.1. Genetic and Epigenetic Markers

Various studies have addressed the relationship between overall tumor mutation load/neoantigen burden and ICB response in NSCLC and melanoma, in the context of anti-PD-1 and anti-CTLA-4 monotherapy [69,70,71,72]. Importantly, neoantigen burden was more strongly associated with tumor immunogenicity than mutation load, reflecting the selection process of the tumor antigens that are finally recognized by the T cell receptors and trigger a sufficient immune activation. Thereby, specific genetic mutations in genes, such as JAK1/2 and BRCA1/2, emerge to predict ICB clinical efficacy, likely due to the failure to activate the IFN-γ target genes and the increased mutation load in tumors deficient in the DNA repair machinery, respectively [37,70,110,123]. Loss-of-function mutations of JAK family members render melanoma resistant to IFN-γ stimulation with insensitivity to IFN-γ-triggered growth arrest [37] and potentially down-regulate PD-L1 expression, which is a possible mechanism for desensitization towards PD-1 blockade [110]. Indeed, melanomas with mutations in IFN-γ signaling are resistant to anti-CTLA-4 blockade [112]; also, this pathway is activated in responders to anti-PD-L1 treatment [124]. In addition, IFN-γ-induced IDO expression is increased in melanoma patients responding to CTLA-4 and PD-L1 blockade [102,124]. Mutations in the DNA double strand repair enzyme BRCA2 result in drastically increased mutational burden, leading to increased responsiveness to PD-1 blockade [70]. In relation with this, mismatch repair deficiency tumors of different origins with germline alterations of MSH2, MSH6, PMS2, or MLH1 were associated with high neoantigen burden, and indicative of recognition by tumor-specific T cells [83]. However, similar mutation and neoantigen profiles were observed in responders and non-responders under ICB treatment [89,110,125]. Other genetic variants that can constitute ICB response biomarkers are the genotypes 1577G/G and CT60G/G in CTLA4, which demonstrate a favorable overall response (OS) in patients receiving anti-CTLA4 therapy [82]. Also, higher TCR clonality identified by sequencing of the β-chain of the TCR is observed in responders to PD-1 blockade rather than CTLA-4 blockade [77,125]. With respect epigenetic biomarkers of response, we reported for the first time a signature of DNA methylation in 301 CpGs, EPIMMUNE, that could be downscaled to the unmethylated state of a single CpG site in the transcription factor FOXP1, which regulates both quiescence in naive CD4^+^ cells [126], and Th follicular cells [127], as predictive of response to ICB in NSCLC patients [28].

#### 5.1.2. Transcriptional Biomarkers

Transcriptional signatures can be also informative of response to PD-1 blockade, and in particular when DNA mutation profiles and immunological features are similar [89]. Several gene expression signatures related to IFN-γ [1,90] and the Wnt/β-catenin signaling pathways [39] have been associated to response to ICB. Other signatures associated with clinical outcomes after ICB introduce novel putative resistance mechanisms such as the action of extracellular matrix components like laminins, which might create a barrier excluding immune cells from penetrating the tumor and thus impairing immunotherapy, or the neutrophil infiltration or activation in progressing [89]. Another recently reported panel of expression markers has proven useful to associate survival advantage with moderate tumor proliferation in comparison with tumors that are highly/poorly proliferative in NSCLC patients treated with ICB [128]. Also, expression of endogenous retroviruses (ERV) RNA is correlated with clinical response to anti-CTLA-4 and PD-L1 treatment [129]. Finally, a novel expression signature based on the overexpression of MAGE-A cancer germline antigens has been reported as a putative specific predictor of resistance to anti-CTLA4 treatment. The expression of these germline antigens is typically restricted to immune-privileged gonadal tissues and several types of tumors, and the MAGE-A types are often targets of anti-tumor T cells in melanoma [91].

#### 5.1.3. Histopathological Biomarkers

At the protein level, histopathological biomarkers include PD-L1 expression, which is a biomarker candidate during anti-PD-1 and PD-L1 monotherapy in the context of melanoma, NSCLC, renal cell carcinoma (RCC), and bladder cancer [130], and several other proteins, in their majority marking the presence of relevant immune cell populations to the efficacy of ICB. PD-L1 so far is the only ICB biomarker for which FDA has approved a companion test for pembrolizumab (anti-PD-1) treatment in patients with NSCLC, gastric or gastroesophageal junction adenocarcinoma, cervical cancer, and urothelial carcinoma (PD-L1 IHC 22C3 pharmDx).

#### 5.1.4. Cellular Biomarkers

Indeed, the immune cell panorama within the tumor differentiates clinical response and resistance in both CTLA4 and PD-1 blockade [131]. PD-1 intratumor CD8^+^ T cell density prior to therapy indicates shrinking radiographic tumor size [77]. More specifically, within the tumor-infiltrating CD8^+^ T cells, the presence of the particular population of CD8^+^ T cells of PD-1^+^CTLA4^+^T cells is associated with progression-free survival (PFS). Melanoma patients with frequency higher than 20% have a PFS of 31.6 months, while those with less than 20% have a PFS of 9.6 months [132]. These cells represent the tumor-associated T cells with a partial exhaustion phenotype; hence, they are more susceptible to reinvigoration via the blockade of the co-inhibitory receptor interaction. On the other hand, the rescue of CD8^+^ T cells alone is not necessarily correlated with clinical response. However, when related to tumor burden, circulating rejuvenated PD-1^+^Ki67^+^CD8^+^ T cells are a better predictor of PFS after PD-1 blockade than rejuvenated cell counts alone [99]. Also, the ratio of CD8^+^ T cells and T_reg_ cells is linearly associated with tumor necrosis in CTLA-4 blockaded melanoma [133]. 

### 5.2. Liquid Biopsy Biomarker Candidates

Circulating components are of great potential for the identification of response biomarkers that can be tested non-invasively and dynamically in bodily fluids [134]. So far, the detection of circulating free DNA (cfDNA) provides clinical guidance for multiple cancer treatment strategies [135]. The mutations identified in cfDNA constitute a reliable surrogate of tumor biopsy, and the increase of post-treatment cfDNA levels might be related to progressive disease in melanoma patients. Moreover, cfDNA levels are informative of response before its clinical manifestation and predict the tumor burden in melanoma patients treated with ICB [136]. Also, copy number instability quantified in cfDNA predicts disease progression and shows better overall accuracy than cfDNA concentration alone in patients with diverse tumors treated with immunotherapy [137]. In addition, a recent proof-of-concept work demonstrated the added value of screening 5mC and 5hmC variants in cfDNA for the diagnosis and prognosis of several types of cancers [138].

Several recent studies also indicate that the detection and quantification of circulating tumor cells (CTCs) can be considered a promising circulating biomarker candidate in ICB. A recently published case report [139] associated the detection of CTCs in peripheral blood with the metastatic process. In addition, PD-L1 was highly expressed in CTCs in advanced head and neck cancer patients, indicating that PD-L1^+^ CTCs could serve as a predictive biomarker of ICB response.

Other proposed biomarkers of response include several proteins and cell populations such as serum level of interleukin-8 (IL-8), which is secreted by the tumor and is inversely correlated with OS in NSCLC and melanoma patients under PD-1 blockade [140]. Serum baseline and post-treatment level of angiopoietin-2 is also inversely related to OS in both anti-CTLA4 and PD-1 therapy [141].

Proteins that belong to the ICR pathways have also been detected in liquid biopsies and are correlated with response; patients with higher ICB pre-treatment soluble PD-L1 levels are more likely to progress, while interestingly the post-treatment increase of PD-L1 level is correlated with partial response in ICB [142]. Co-inhibitory IC T cell immunoglobulin mucin 3 (TIM3) and PD-1 and IL-15 serum level are negatively associated with long survival after CTLA-4 blockade, in which context, IL-15 increases TIM3 and PD-1 expression [143]. 

With regards to circulating immune populations, it has been reported that the amount of circulating PD-1^+^ CD4^+^T_eff_ cells is inversely related to OS in anti-CTLA-4 treated prostate cancer patients, with no significant difference in the case of PD-1^+^ CD8^+^ T cells [144]. Also, pre-treatment levels of circulating CD45RO^+^CD8^+^ T cells are positively correlated with patient survival after CTLA-4 blockade. In addition, a higher fraction of circulating CD4^+^ICOS^hi^ T cells indicates longer survival after CTLA-4 blockade [145]. Low baseline LDH level, high relative/absolute eosinophil counts, and relative lymphocyte counts are correlated with prolonged OS in melanoma anti-PD-1 and CTLA-4 treatment [146,147]. Finally, possibly because the BIM level positively reflects PD-1 expression and PD-1/PD-L1 interaction, an increased frequency of circulating Bim^+^PD-1^+^CD8^+^ T cell has been found to be correlated with anti-PD-1 efficacy [101]. 

## 6. DNA Methylation and Hydroxymethylation as Potential Biomarkers of Response to Cancer Immunotherapy

### 6.1. Involvement of DNA Methylation and Hydroxymethylation in Tumor Immune Evasion

The fact that 5mC and 5hmC are dynamic marks that correlate with tumor immune evasion and T cell exhaustion opens new avenues for clinical biomarkers research, where epigenetic variants or signatures can represent a new class of biomarkers for ICB response. 

In the context of T cell exhaustion, it can be hypothesized that DNA methylation can be involved in the maintenance and reinforcement of exhaustion gene expression signatures. Indeed, a progressive Dnmt3a-mediated de novo methylation has been observed in murine antigen-specific CD8 T cells that underwent exhaustion via repression of key genes implicated in the effector function, the proliferation, metabolism, and tumor recruitment of immune cells, impairing T cell expansion and clonal diversity under treatment with anti-PD-1 [148]. These findings are corroborated by observations in chronic lymphocytic choriomeningitis virus (LCMV)-infected mice, where the preservation of a specific chromatin configuration was associated with a transient reinvigoration of T cells induced by treatment with PD-1 [149]. This reinvigoration was likely mediated mainly by NFκB signaling, and the preserved chromatin configuration that sustains the transient nature of the anti-tumor immune reactivation could be related to the post-treatment static expression of key transcription factors implicated in the exhaustion phenotype such as T-bet and Eomes [149]. Indeed, in primary human CD4+ T cells that were TCR-stimulated in vitro using antibodies against CD3 and CD28, the genomic binding regions of specific enhancers and transcription factors involved in the activation of the T cells overlap with regions of accessible chromatin in the post-treatment remodeled scenario [150]. Interestingly, in some individuals, these regions have mutations associated with autoimmune diseases and enhancer specific of different T cells. In addition, correlations between specific SNPs and local regions of accessible chromatin have been delineated, raising the possibility that interindividual genetic variation is affecting the chromatin remodeling after treatment with ICB [150]. Combined, the highlighted studies provide evidence of an important role of epigenetic mechanisms in relapse risk of patients post ICB therapy [151,152]. 

The involvement of DNA methylation in the transcriptional reprogramming of the T cells has also been reported in the context of T cell exhaustion following viral infections, where the PD-1 promoter undergoes extensive de-methylation resulting in permanent CD8^+^T cell exhaustion [153]. By contrast, in the acute setting, it is subsequently re-methylated in the transition of T_eff_ to T_mem_ [153]. Ten-eleven translocation (TET) dioxygenases-dependent oxidation of DNA methylation has been related to reprogramming processes in differentiated cells [154]. Indeed, this form of active de-methylation is underlying the dynamics of 5mC and 5hmC in the promoter of the murine gene coding for PD-1 (Pdcd1) in CD4+ autoimmune T effector cells. The deposition of 5hmC seems to mark a poised state. Only in the context of permanent induction of PD-1 provoked by peptide immunotherapy, 5hmC is erased in mouse [155]. In view of this information, 5hmC constitutes and good candidate for monitoring the phenotypic reprogramming of T_eff_ cells during exhaustion or ICB resistance.

Regarding the tumor-intrinsic reprogramming that features the immunosuppressive TME, it has been reported that DNA methylation at the promoter regions of the tumor Th1-type chemokines CXCL9 and CXCL10, mediated by DNMT1, represses their transcript and protein expression in ID8 ovarian cancer in C57/BL6 mice. Consequently, cytotoxic T cell trafficking into the tumor microenvironment decreases. Interestingly, the epigenetic modulation of the expression of these chemokines with azacytidine indicated that the modification of the epigenetic program can improve the T_eff_ infiltration as well as the response to anti-PD-L1 agents [156]. Another tumor-intrinsic adaptation against the immune anti-tumor activity is the DNA methylation-induced repression of tumor-specific antigens [157]. For example, promoter hypermethylation of cancer/testis antigens abrogates the tumor immunogenicity by nullifying the recognition and response of antigen-specific CD8+ T cells [158,159,160]. On the other hand, de-methylation increases the level of endogenous retrovirus double-stranded RNA and triggers the activation of the MDA5/MAVS signaling pathway, which stimulates immune-related transcription factors and IFN response, and reduces the tumor growth [161,162].

As highlighted above, epigenetic alterations associated with immune response and evasion in immune cells are extensive, and they set the stage for the identification of 5mC and 5hmC biomarkers of response. Specifically, Tet2 controls the differentiation of naïve CD4^+^ T cells into several lineages of helper T (Th) cells in mice, thereby directly modulating cytokine production [163]. Moreover, Tet2 has been found to contribute to CD8+ T-lymphocyte effector differentiation [164]. The important role of TET-mediated active de-methylation is furthermore exemplified by its direct control of Foxp3 expression in T_reg_, where demethylation gives rise to lineage-specific epigenetic signatures that guide development and maturation of Foxp3^+^ T_reg_ within the thymus [165]. In addition, TET activity modulation is related to the maintenance of Foxp3 expression [166], and active de-methylation of the IL2 promoter coincides with increased IL2 expression upon CD4^+^ cell activation [167].

Even though the field is prolific in determining the roles of 5mC and 5hmC profiles in different tumor immune evasion scenarios, it is important to emphasize that the mechanisms underlying these associations are, so far, poorly understood. Scharer and collaborators identified a step-wise differentiation process of CD8^+^ T cells triggered by antigen presentation, in which inactive genes, such as Pdcd1 in naïve cells, were progressively demethylated towards CD8^+^ T_eff_-cells [168]. These transitions would start by the DNA binding of transcription factors that do not contain any CpGs in their binding site (e.g., NFATc1). They would induce histone H3 and H4 acetylation, as well as DNA de-methylation. The generated open chromatin landscape is permissive to the binding of DNA methylation-sensitive transcription factors that direct the rewiring of the expression towards the effector phenotype (e.g., Pdcd1). Interestingly, the DNA methylation-sensitive transcription factors c-JUN, JUND, c-MYC, CREB/ATF, CTCF, and ETS1 are expressed ubiquitously in differentiating CD8+ T cells [169].

### 6.2. Emerging Evidence Supporting the Roles of DNA Methylation and Hydroxymethylation as Epigenetic Predictors of ICB Response

The appreciation of the important role of the 5mC and 5hmC landscape has given rise to the emerging discipline of pharmacoepigenetics. Particularly in tumor cells, epigenomic patterns undergo substantial changes, which has resulted in the discovery of an increasing repertoire of epigenomic biomarkers. For an overview of this field, we refer the interested reader to recent comprehensive reviews [170,171,172,173,174]; however, we would like to emphasize how such epigenetic alterations, when associated to ICB response, could serve useful for monitoring the clinical benefit during the course of the disease by agglutinating in Table 4 the most relevant DNA methylation non-invasive cancer biomarkers. It is important to note that the great majority of identified cytosine methylation biomarkers, so far, pertain to DNA methylation, at least in part because methodologies that discriminate 5mC from 5hmC have only been recently developed [175,176]. By adapting the newly arising technologies that allow distinct typing of 5mC and 5hmC, we have characterized the absorption, distribution, metabolism, and excretion (ADME)-related methylome and hydroxymethylome of the human liver [177], and we have reported a proof-of-principle study where specific 5hmC mapping unmasks a unexpectedly high degree of hypermethylation in human hepatocellular carcinoma tumors and contributes to the identification of novel diagnostic biomarkers [178]. 

Concerning the epigenetic biomarkers of response to ICB, we have recently reported the association of the methylation state of 301 CpGs that conform to the “EPIMMUNE” signature, and its subrogation to the unmethylated state of a single CpG of *FOXP1*, a transcription factor involved in the regulation of quiescent CD4^+^ cells, and the regulation of follicular T helper cells, with overall and progression-free survival to anti-PD-1 treatment in NSCLC patients [28]. We speculate that the release of the immunosuppression that had been induced by the PD-1/PD-L1 interaction would give rise to the activation of a remaining pool of naïve CD4^+^ cells and the subsequent enhancement of the anti-tumor immune activity. Interestingly, the most amply studied response predictors, such as CD8, PD-L1 immunodetermined proteins, and the tumor mutational burden, did not separate significantly those patients with better treatment outcome. This constitutes the first reported association of epigenetic variants with the clinical benefit of ICB.

To our knowledge, so far, no specific 5hmC biomarker of response to cancer therapy has been validated, although several lines of evidence suggest the involvement of TET enzymes in the response mechanisms; for example, TET1 knockdown in lung cancer cell lines with EFGR mutations leads to enhanced EFGR inhibitor resistance, while the responsive tumors show increased TET1 expression [179]. As discussed above, epigenetic remodeling of 5mC and 5hmC signatures controls many aspects of the reprogramming events associated with innate and acquired resistance to ICB, both tumor-intrinsic and extrinsic. Indeed, DNA methylation seems to control PD-1, PD-L1, PD-L2, and CTLA-4 gene expression; when these genes are silenced, the antigen presentation and the immune cytotoxic effects are inhibited [180,181]. The hypermethylation-derived silencing of CTLA-4 and PD-1 was also observed in baseline tumor biopsies compared to their pair-matched tissues in NSCLC patients [87]. In addition, in colorectal cancer, PD-L1 expression is associated with CpG island hypermethylation in a subpopulation of BRAF V600E carriers with high infiltration of CD3^+^ T cells [182]. Moreover, in metastatic melanoma patients treated with CTLA-4 blockers, responders and non-responders have a differential pattern of DNA methylation in specific genes of the nervous system development and neuron differentiation pathways [88]. Because neuron and melanocytes have precursor cells of neural crest origin, these results suggest a process of de-differentiation of the transformed melanocytes refractory to ICB treatment. Noteworthy, de-differentiation induced by inflammation has been already reported as a possible tumor immune evasion mechanism [183]. 

Stepwise hypermethylation has also been related to the facilitation of tumor escape by repressing expression of the IFN regulator IRF8 [184]. Importantly, the positive effect of de-methylation on transcriptional activity for some immune-related genes, including PD-L1 and genes of the interferon signaling cascade, has been validated in vitro, corroborating that epigenetic modulation might be a useful tool to sensitize patients to anti-PD-L1 ICB, and the facilitation of tumor escape by repressing IFN regulatory factor 8 transcriptional expression [185]. Importantly, the positive effect of de-methylation on transcriptional activity for some immune-related genes, including PD-L1 and genes of the interferon signaling cascade, has been validated in vitro, corroborating that epigenetic modulation might be a useful tool to sensitize patients to anti-PD-L1 ICB [186]. In addition, in a mouse ovarian cancer model, de-methylation triggers the type I interferon signaling pathway, sensitizing mice to anti-CTLA4 therapy [161]. Furthermore, azacytidine and CTLA-4 mAb combination therapy represses tumor growth more strongly than each of the monotherapies, via the upregulation of MHC class I components as the putative mechanism [187]. Other studies also identify the increase of the lymphocyte infiltration and the T helper 1-type chemokines and cytokines as possible cause of the better observed outcome when de-methylation and anti-PD-L1 and CTLA4 agents are used in combination in a murine ovarian cancer model [188]. Interestingly, the inverse interaction between immune signaling pathways and epigenetic regulation in cancer has also been observed, where NF-kβ interacts with TET1 promoter for its downregulation in breast cancer cells [189].

As a consequence of this ample pre-clinical evidence and the appreciation that epigenetic reprogramming participates in acquired drug resistance, there is a drastic increase of clinical trials that explore the synergy of epidrug combination therapies [170,190]. Indeed, demethylating agents and histone deacetylases are being combined with ICB in numerous clinical trials and types of malignancies. Sun and collaborators have recently reviewed the current clinical trials that combine histone modifications inhibitors with immunotherapy. The majority of combinations include anti-PD-1 drugs and histone deacetylases. Some suggested mechanisms for the synergy of the combinations in enhancing the response and preventing the relapse are the upregulation by inhibitors of histone deacetylases of CD80 and CD86 in the context of anti-CTLA-4 treatment, the regulation of immune checkpoint ligands, and the induction of tumor neoantigens on tumor cells for PD-1/PD-L1 therapy. Consistently, most of the combinatorial strategies with DNA-demethylating and histone modification inhibiting drugs aim at the upregulation of tumor neoantigens and the downregulation of PD-L1 expression. Also, BET/bromodomain 4 inhibitors promote depolarization of macrophages into immunostimulatory ones, leading to the decrease of MDSCs in the tumor microenvironment [191].

## 7. Future Perspectives and Conclusions

The reactivation of the anti-tumor immune response with antibodies that compete with the co-inhibitory immune receptor could be intuitively considered an Achilles heel for tumor immunoediting and evasion from immune surveillance. However, given the absence of good response biomarkers and the complex network of interactions of the TME that influences the efficacy of ICB, a significant fraction of patients experience innate and acquired resistance, and some even hyperprogression. As we have extensively described in this review, much effort has been devoted to the identification of biomarkers that could predict response to ICB. Lately, new approaches use top-down strategies and Next Generation Sequencing to identify novel tumor-intrinsic and -extrinsic mechanisms. However, despite the importance of epigenetic regulation for reprogramming events during tumor immune evasion, only a single study has reported the identification of CpG-site specific epigenetic biomarkers of response to ICB in human samples [28]. Furthermore, DNA methylation stands as the putative mechanism for the maintenance of the exhaustion gene expression program during ICB. Therefore, we anticipate that the search for 5mC and 5hmC signatures associated with differential clinical outcomes of ICB will reveal new biomarkers and give rise to novel mechanistic hypotheses that could be integrated in multiomics prediction algorithms to further personalize cancer immunotherapy.

## Figures and Tables

**Table 1:** Updated FDA approved immune checkpoint inhibitors and their indications.

Drug	Approval Date	Mechanism	Sample Size	Reference Clinical Trial	Cancer Type	Indications
Ipilimumab (YERVOY®) *	28/10/2015	CTLA4	951	EORTC (NCT00636168)	Melanoma	Adjuvant treatment of cutaneous melanoma patients with pathologic involvement of regional lymph nodes of more than 1 mm who have undergone complete resection
Ipilimumab (YERVOY®) *	25/3/2011	CTLA-4	676	MDX010-20 (NCT00094653)	Melanoma	Unresectable or metastatic melanoma with previous systematic treatment previously
Pembrolizumab (KEYTRUDA®) *	04/09/2014	PD-1	173	KEYNOTE-001 (NCT01295827)	Melanoma	Unresectable or metastatic melanoma and disease progression following Ipilimumab and, if *BRAF* V600 mutation positive, a BRAF inhibitor
Pembrolizumab (KEYTRUDA®) *	18/12/2015	PD-1	834+540	KEYNOTE-006 (NCT01866319); KEYNOTE-002 (NCT01704287)	Melanoma	Unresectable or metastatic melanoma
Nivolumab + Ipilimumab (OPDIVO® + YERVOY®) *	30/09/2015	PD-1, CTLA4	142	CheckMate-069 (NCT01927419)	Melanoma	*BRAF* V600 wild-type, unresectable or metastatic melanoma
Nivolumab (OPDIVO®) *	22/12/2014	PD-1	120	CheckMate-037 (NCT01721746)	Melanoma	Unresectable or metastatic melanoma and disease progression following Ipilimumab and, if *BRAF* V600 mutation positive, a BRAF inhibitor
Pembrolizumab (KEYTRUDA®) *	15/02/2019	PD-1	1019	KEYNOTE-054 (NCT02362594)	Melanoma	Melanoma with involvement of lymph node(s) following complete resection
Nivolumab (OPDIVO®) *	20/12/2017	PD-1	906	CheckMate-238 (NCT02388906)	Melanoma	Adjuvant treatment of advanced melanoma
Nivolumab + Ipilimumab (OPDIVO® + YERVOY®)	16/04/2018	PD-1, CTLA4	847	CheckMate-214 (NCT02231749)	Hepatocellular carcinoma	Intermediate or poor risk advanced hepatocellular carcinoma without prior treatment
Pembrolizumab (KEYTRUDA®)	09/11/2018	PD-1	104	KEYNOTE-224 (NCT02702414)	Hepatocellular carcinoma	Hepatocellular carcinoma previously treated with Sorafenib
Nivolumab (OPDIVO®)	22/09/2017	PD-1	154	CheckMate-040 (NCT01658878)	Hepatocellular carcinoma	Hepatocellular carcinoma previously treated with sorafenib
Pembrolizumab (KEYTRUDA®) *	15/03/2017	PD-1	210	KEYNOTE-087 (NCT02453594)	Lymphoma	Refractory classical Hodgkin lymphoma patients, or those who have relapsed after three or more prior lines of therapy
Nivolumab (OPDIVO®) *	17/05/2016	PD-1	95	CheckMate-205 (NCT02181738); CheckMate-039 (NCT01592370)	Lymphoma	Recurrent Hodgkin lymphoma following autologous hematopoietic stem cell transplantation and post-transplantation Brentuximab Vedotin
Pembrolizumab (KEYTRUDA®)	13/06/2018	PD-1	53	KEYNOTE-170 (NCT02576990)	Lymphoma	Refractory primary mediastinal large B-cell lymphoma patients, or who have relapsed after two or more prior lines of therapy
Cemiplimab-rwlc (LIBTAYO®) *	28/09/2018	PD-1	108	R2810-ONC-1423 (NCT02383212) R2810-ONC-1540 (NCT02760498)	Cutaneous squamous cell carcinoma	Metastatic or locally advanced cutaneous squamous cell carcinoma patients who are not candidates for curative surgery or curative radiation
Pembrolizumab (KEYTRUDA®) *	05/08/2016	PD-1	174	KEYNOTE-012 (NCT01848834)	Squamous cell carcinoma of the head and neck	Recurrent or metastatic squamous cell carcinoma of the head and neck with progression on or after platinum-containing chemotherapy
Nivolumab (OPDIVO®) *	10/11/2016	PD-1	361	CheckMate-141 (NCT02105636)	Squamous cell carcinoma of the head and neck	Advanced squamous cell carcinoma of the head and neck with progression on/after a platinum-based therapy
Nivolumab (OPDIVO®)	31/07/2017	PD-1	74	CheckMate-142 (NCT02060188)	Colorectal	Treatment of patients 12 years and older with mismatch repair deficient and microsatellite instability high metastatic colorectal cancer that has progressed following treatment with Fluoropyrimidine, Oxaliplatin, and Irinotecan
Nivolumab + Ipilimumab (OPDIVO® + YERVOY®)	10/07/2018	CTLA4	82	CheckMate-142 (NCT02060188)		Metastatic colorectal cancer with high microsatellite instability or mismatch repair deficiency
Pembrolizumab (KEYTRUDA®)	23/05/2017	PD-1	149	KEYNOTE-016 (NCT01876511); KEYNOTE-164 (NCT02460198); KEYNOTE-012 (NCT01848834); KEYNOTE-028 (NCT02054806); KEYNOTE-158 (NCT02628067)	Colorectal	Unresectable or metastatic, microsatellite instability-high or mismatch repair deficient solid tumors patients that have progressed following prior treatment and who have no satisfactory alternative treatment options or with microsatellite instability-high or mismatch repair deficient colorectal cancer that has progressed following treatment with Fluoropyrimidine, Oxaliplatin, and Irinotecan
Pembrolizumab (KEYTRUDA®)	12/06/2018	PD-1	98	KEYNOTE-158 (NCT02628067)	Cervical	Recurrent or metastatic cervical cancer patients with progression on or after chemotherapy whose tumors express PD-L1 as determined by an FDA-approved test
Pembrolizumab (KEYTRUDA®) *	11/04/2019	PD-1	1274	KEYNOTE-042 (NCT02220894)	Lung	First-line treatment of patients with stage III non-small-cell lung cancer who are not candidates for surgical resection or definitive chemoradiation or metastatic non-small cell lung cancer. Patients’ tumors must have no *EGFR* or *ALK* genomic aberrations and express PD-L1 (Tumor Proportion Score [TPS] ≥1%) determined by an FDA-approved test
Atezolizumab (TECENTRIQ®) + chemotherapy *	06/12/2018	PD-L1	1202	IMpower150 trial (NCT02366143)	Lung	Metastatic non-squamous, non-small-cell lung cancer with no *EGFR* or *ALK* genomic tumor aberrations
Atezolizumab (TECENTRIQ®) *	18/10/2016	PD-L1	1137	POPLAR (NCT01903993); OAK (NCT02008227)	Lung	Metastatic non-small-cell lung cancer patients whose disease progressed during or following platinum-containing chemotherapy.
Pembrolizumab (KEYTRUDA®) + pemetrexed and carboplatin *	10/05/17	PD-1	123	KEYNOTE-021 (NCT02039674)	Lung	Previously untreated metastatic non-squamous non-small-cell lung cancer
Nivolumab (OPDIVO®) *	09/10/2015	PD-1	582	CheckMate-057 (NCT01673867)	Lung	Metastatic non-small-cell lung cancer with progression on or after platinum-based chemotherapy
Pembrolizumab (KEYTRUDA®) + carboplatin/ paclitaxel *	30/10/2018	PD-1	559	KEYNOTE-407 (NCT02775435)	Lung	Metastatic squamous non-small cell lung cancer
Pembrolizumab (KEYTRUDA®) *	24/10/2016	PD-1	305 + 1033	KEYNOTE-024 (NCT02142738); KEYNOTE-010 (NCT01905657)	Lung	Metastatic non-small-cell lung cancer patients whose tumors express PD-L1 as determined by an FDA-approved test
Nivolumab (OPDIVO®) *	04/03/2015	PD-1	272	CheckMate-017 (NCT01642004)	Lung	Metastatic squamous non-small-cell lung cancer with progression on or after platinum-based chemotherapy
Pembrolizumab (KEYTRUDA®) + pemetrexed and platinum *	20/08/2018	PD-1	616	KEYNOTE-189 (NCT02578680)	Lung	Metastatic, non-squamous non-small-cell lung cancer, with no with no *EGFR* or *ALK* genomic tumor aberrations
Durvalumab (IMFINZI®) *	06/02/2018	PD-L1	713	PACIFIC (NCT02125461)	Lung	Unresectable stage III non-small cell lung cancer patients whose disease has not progressed following concurrent platinum-based chemotherapy and radiation therapy
Pembrolizumab (KEYTRUDA®) *	02/10/2015	PD-1	61	KEYNOTE-001 (NCT01295827)	Lung	Metastatic non-small cell lung cancer patients whose tumors express programmed death ligand 1 as determined by an FDA-approved test, with disease progression on or after platinum-containing chemotherapy
Atezolizumab (TECENTRIQ®) + carboplatin and etoposide *	18/03/2019	PD-L1	403	IMpower133 (NCT02763579)	Lung	Extensive-stage small cell lung cancer
Nivolumab (OPDIVO®)	16/08/2018	PD-1	109	CheckMate-032 (NCT01928394)	Lung	Progressive metastatic small cell lung cancer with progression after platinum-based chemotherapy and other lines of therapy
Nivolumab (OPDIVO®) *	02/02/2017	PD-1	270	CheckMate-275 (NCT02387996)	Urothelial	Locally advanced or metastatic urothelial carcinoma patients who have disease progression during or following platinum-containing chemotherapy or have disease progression within 12 months of neoadjuvant or adjuvant treatment with a platinum-containing chemotherapy
Durvalumab (IMFINZI®)	01/05/2017	PD-L1	182	Study 1108 (NCT01693562)	Urothelial	Locally advanced or metastatic urothelial carcinoma patients who have disease progression during or following platinum-containing chemotherapy or who have disease progression within 12 months of neoadjuvant or adjuvant treatment with platinum-containing chemotherapy
Atezolizumab (TECENTRIQ®) *	18/05/2016	PD-L1	310	IMvigor210 (NCT02108652)	Urothelial	Locally advanced or metastatic urothelial carcinoma patients who have disease progression during or following platinum-containing chemotherapy or have disease progression within 12 months of neoadjuvant or adjuvant treatment with platinum-containing chemotherapy
Avelumab (BAVENCIO®)	09/05/2017	PD-L1	242	JAVELIN Solid Tumor (NCT01772004)	Urothelial	Locally advanced or metastatic urothelial carcinoma patients whose disease progressed during or following platinum-containing chemotherapy or within 12 months of neoadjuvant or adjuvant platinum-containing chemotherapy
Pembrolizumab (KEYTRUDA®) *	18/05/2017	PD-1	542	KEYNOTE-045 (NCT02256436)	Urothelial	Locally advanced or metastatic urothelial carcinoma patients who have disease progression during or following platinum-containing chemotherapy or within 12 months of neoadjuvant or adjuvant treatment with platinum-containing chemotherapy
Pembrolizumab (KEYTRUDA®)	19/12/2018	PD-1	50	KEYNOTE-017 (NCT02267603)	Merkel cell carcinoma	Recurrent locally advanced or metastatic Merkel cell carcinoma
Avelumab (BAVENCIO®) *	23/3/2017	PD-L1	1738	JAVELIN Merkel 200 (NCT02155647)	Merkel cell carcinoma	Metastatic Merkel cell carcinoma
Nivolumab (OPDIVO®) *	23/11/2015	PD-1	821	CheckMate-025 (NCT01668784)	Renal	Advanced renal cell carcinoma in patients with previous anti-angiogenic therapy
Atezolizumab (TECENTRIQ®) *	08/03/2019	PD-L1	902	IMpassion130 (NCT02425891)	Breast	Unresectable locally advanced or metastatic triple-negative breast cancer patients whose tumors express PD-L1 (PD-L1 stained tumor-infiltrating immune cells [IC] of any intensity covering ≥ 1% of the tumor area), as determined by an FDA-approved test
Pembrolizumab (KEYTRUDA®)	22/09/2017	PD-1	259	KEYNOTE-059 (NCT02335411)	Gastric/gastro-esophageal junction	Recurrent locally advanced or metastatic, gastric or gastroesophageal junction adenocarcinoma patients whose tumors express PD-L1 as determined by an FDA-approved test

* Drug administration also approved by the European Medicines Agency (EMA) for the same cancer type.

**Table 2:** Active clinical trials of immune checkpoint blockade for novel oncological indications.

Drug	Targeted IC	Sample Size	Cancer Type	Response Rate	Phase	Trial Number
Ipilimumab	CTLA-4	100	Melanoma (stage III/IV)	10.9%	III/IV	NCT00094653
Pembrolizumab	PD-1	31	Hodgkin Lymphoma (recurred)	65%	I	NCT01953692
Pembrolizumab	PD-1	26	Locoregional Merkel-cell carcinoma (advanced)	56%	II	NCT02267603
Nivolumab	PD-1	240	Squamous-Cell Carcinoma (relapsed or advanced)	13.3%	III	NCT02105636
Nivolumab	PD-1	410	Renal-Cell Carcinoma (advanced)	25%	III	NCT01668784
Pembrolizumab	PD-1	270	Urothelial Carcinoma (advanced)	21.1%	III	NCT02256436
Pembrolizumab	PD-L1	27	Triple-Negative Breast Cancer (advanced)	18.5%	I	NCT01848834
Nivolumab	PD-1	39	Hepatocellular carcinoma (advanced)	23%	I/II	NCT01658878
MDX1105-01 (anti–PD-L1)	PD-L1	207	Non-small-cell lung cancer, melanoma, colorectal cancer, renal cell carcinoma, prostate cancer, ovarian cancer, gastric cancer, breast cancer	12.6%	I	NCT00729664
Atezolizumab	PD-L1	175	Non-small-cell lung cancer, renal cell carcinoma, melanoma, other tumors	18%	I	NCT01375842
Tremelimumab	CTLA-4	17	Hepatocellular carcinoma (advanced with chronic hepatitis C)	17.6%	II	NCT01008358
Avelumab	PD-L1	88	Merkel cell carcinoma (chemotherapy-refractory stage IV)	31.8%	II	NCT02155647
Atezolizumab	PD-L1	116	Triple-negative breast cancer (metastatic)	9.5%	I	NCT01375842
Atezolizumab	PD-L1	32	Head and neck cancer	22%	I	NCT01375842
Atezolizumab	PD-L1	95	Urothelial cancer (metastatic)	26%	I	NCT01375842
Nivolumab	PD1	296	Melanoma (advanced), non–small-cell lung cancer, prostate cancer (castration-resistant), renal-cell cancer, colorectal cancer	18% in non-small-cell lung cancer, 28% in melanoma, 27% in renal-cell cancer	I	NCT01354431
Pidilizumab	PD-1	66	Diffuse large B-cell lymphoma	51%	II	NCT00532259
Pidilizumab	PD-1	32	Follicular lymphoma (relapsed)	66%	II	NCT00904722
Nivolumab	PD-1	23	Hodgkin’s lymphoma (relapsed or refractory)	87%	I	NCT01592370
Lambrolizumab	PD-1	135	Melanoma (advanced)	38%	I	NCT01295827
Nivolumab	PD1	107	Melanoma (advanced)	30.8%	I	NCT00730639
Nivolumab	PD1	418	Melanoma (untreated without BRAF mutation)	40.0%	III	NCT01721772
Nivolumab	PD1	631	Melanoma (advanced that progressed after anti-CTLA-4 treatment)	31.7%	III	NCT01721746
Pembrolizumab	PD1	495	Non–small-cell lung cancer	19.4%	I	NCT01295827
Nivolumab	PD1	272	Squamous-cell non-small-cell lung cancer (advanced)	20%	III	NCT01642004
Nivolumab	PD1	129	Non–small-cell lung cancer (previously treated advanced)	17%	I	NCT00730639

**Table 3:** Candidate response biomarkers for immune checkpoint blockade.

Biomarker	Type	Target of the Test	Cohort Size	Predictive Power	Assay/Predictive Value
Amount and clonality of TCR repertoire	Genetic	Immune	25	*p* = 0.004	TCR sequencing In metastatic melanoma, high clonality of TCR repertoire significantly correlated with clinical response to pembrolizumab treatment [77]
Tumor neoantigen clonality	Genetic	Tumor	139	No ITH threshold, HR = 0.47, *p* = 0.025 ITH threshold = 0, HR = 0.212, *p* = 0.019 ITH threshold = 0.01, HR = 0.33, *p* = 0.008 ITH threshold = 0.05, HR = 0.45, *p* = 0.083	Whole exome sequencing In melanoma patients treated with ipilimumab or tremelimumab, overall survival was significantly better in tumors with low neoantigen intratumor heterogeneity (ITH) and high clonal neoantigen burden [68]
Tumor mutational burden (TMB)	Genetic	Tumor	16, 49	HR = 0.19, *p* = 0.01, HR = 1.38, *p* = 0.24	Whole exome sequencing targeted next generation sequencing High TMB associated with clinical benefit [71,78,79]
ctDNA	Genetic	Tumor	28	Progression-free survival, HR = 0.29, *p* = 0.03 Overall survival, HR = 0.17, *p* = 0.007	ctDNA level by next-generation sequencing High value of ctDNA drop indicates good response [80]
*JAK1, JAK2*	Genetic	Immune	4	/	*JAK1/JAK2* mutation by whole genome sequencing *JAK1/2* mutation indicates bad response [37,39,81]
*β2 microglobulin (B2M)*	Genetic	Tumor	40, 34	*p* = 0.009, *p* = 0.004	B2M mutation by whole-genome sequencing B2M mutation indicates bad response [69]
Germinal SNPs −1577G/G and CT60G/G in *CTLA4*	Genetic	Germinal	173	−1577G>A, OR = 0.04 and 0.24 CT60G>A, OR = 0.07 and 0.28	SNPs by genotyping. −1577G>A and CT60G>A indicates good response [82]
*BRCA1/2*	Genetic	Tumor	38	OR = 6.2, *p* = 0.002	*BRCA2* mutation by whole-genome sequencing. *BRCA2* mutation indicates good response [70,83,84]
*KRAS, TP53*	Genetic	Tumor	54 (immunotherapy cohort)	pTP53 mut = 0.042 pKRAS mut = 0.003	*TP53* and *KRAS* mutation by whole genome sequencing *TP53/KRAS* mutation indicates good response [85]
*MDM2, EFGR*	Genetic	Tumor	155	OR (*MDM2*) = 10.8 OR (*EGFR*) = 8.36	Targeted sequencing. *MDM2/EGFR* amplification indicates bad response [75]
rs17388568	Genetic	Germinal	169	OR = 0.26, *p* = 0.0002	Genotyping by Sequenom MassArray. rs17388568 associated with response [86]
*FOXP1* BS-5mC	Epigenetic	Immune	61	Progression-free survival, HR = 0.415, *p* = 0.0063 Overall survival, HR = 0.409, *p* = 0.0094	*FOXP1* methylation by EPIC array and pyrosequencing *FOXP1* methylation indicates bad response [28]
*CTLA4, PDCD1*	Epigenetic	Tumor	18	*p* < 0.01	Array-based CpG-methylation assessment Significant differences in the CpG-methylation patterns between tumor tissues and matched controls were observed [87]
68 genes	Epigenetic	Tumor	18	*p* < 0.05	Differential DNA methylation pattern between durable clinical benefit vs. no clinical benefit [88]
*LAMA3*	Transcriptional	Tumor	26	*p* = 0.003	RT-PCR In patients with metastatic melanoma, *LAMA3* is differentially expressed in regressing versus progressing metastases [89]
IFN-γ-associated gene-expression score	Transcriptional	Tumor	19, 62, 43, 33	*p* < 0.05	Expression score by NanoString gene expression profiling High value of expression level indicates better response [1,90]
*KRT1, KRT5, KRT10, KRT15, KRT78* (keratin genes) *LOR, FLG2, DSC1, DSC3, LGALS7, LAMA3, KLK7* (cell adhesion genes) *WNT3, WNT5A* (Wnt pathway genes)	Transcriptional	Immune/tumor	10	FC ≥ 1.5	Gene expression by whole genome microarray High values indicate bad response [89]
Melanoma Antigen Gene (MAGE)-A cancer-germline antigens	Transcriptional/histopathological	Tumor	55	*p* = 0.011	Expression of MAGE-A cancer-germline antigens by RT-PCR and IHC. High value indicates bad response [91]
PD-L1	Histopathological	Immune/tumor	455, 305, 26	Overall survival, *p* = 0.06 (≥1% PD-L1), *p* < 0.001 (≥5% and ≥10% PD-L1), Progression-free survival, *p* = 0.02 (≥1% PD-L1), *p* < 0.001 (≥5% and ≥10% PD-L1), Objective response rate, *p* = 0.002 (≥1%, ≥5% and ≥10% PD-L1); Overall survival HR for death, 0.60, *p* = 0.005; *p* = 0.006.	PD-L1 IHC In advanced non-small-cell lung cancer patients treated with Nivolumab, PD-L1 expression predicts overall survival, progression-free survival, and objective response rate, with increasing interaction p-values with increasing % of PD-L1 expression [92] In PD-L1 negative metastatic non-small-cell lung cancer patients, ICB efficacy is equivalent to chemotherapy [93] In advanced non-small-cell lung cancer, first line setting pembrolizumab in monotherapy is correlated with better progression-free survival (PFS) and overall survival (OS) than platinum-doublet chemotherapy, only if PD-L1 expression is equal to or above 50% [94] In metastatic melanoma treated with Pembrolizumab, the responders presented significantly higher numbers of PD-L1+ cells when compared to the patients that progressed (*p* = 0.006) [77]
CD8	Histopathological	Immune	46	*p* < 0.0001	CD8 IHC In metastatic melanoma treated with Pembrolizumab, the responders presented significantly higher numbers of CD8+ cells when compared to the patients that progressed [77]
PD-1	Histopathological	Immune	41	*p* = 0.0002	PD-1 IHC In metastatic melanoma treated with Pembrolizumab, the responders presented significantly higher numbers of PD-1+ compared to the patients that progressed [77]
Immunoscore	Histopathological	Immune	475	Disease-specific survival, HR = 2.4 (microsatellite instable) Overall survival, HR = 1.8 (microsatellite instable) Disease-specific survival, HR = 3.4 (microsatellite stable) Overall survival, HR = 2.43 (microsatellite stable)	CD3 and CD8 or CD8 and CD45RO IHC In colorectal cancer patients treated with anti-PD-1, immunoscore is a better response biomarker than microsatellite instability. Multivariate analysis shows a significant correlation of Immunoscore with disease-specific survival, disease-free survival, and overall survival despite their microsatellite status [95]
CD63, E-cadherin, CXCL4, CXCL12	Histopathological /protein	Immune/tumor	8	pCD63 = 0.013 pE-cadherin = 0.005 pCXCL4 = 0.04 pCXCL12 = 0.041	CD63, E-cadherin by IHC, CD63, E-cadherin, CXCL4, CXCL12 by proteomics All of them indicate better response [96]
PTEN	Histopathological	Tumor	39	*p* = 0.029	PTEN IHC High value indicates bad response (*p* = 0.029) [97]
Circulating CD8^+^ T cells	Cellular	Immune	43	% survival, HR = 0.21, *p* = 0.00063	Circulating CD8+ T cells by flow cytometry. High value indicates response [98]
Circulating monocytic MDSCs (CD14+)	Cellular	Immune	43	Overall survival, HR = 2.89, *p* = 0.002203	Circulating monocytic MDSCs (CD14+) by flow cytometry. High value indicates bad response [98]
Circulating PD-1^+^ CD8^+^ T cells	Cellular	Immune	25	*p* = 0.02	Circulating PD-1+ CD8+ T cells by flow cytometry High value indicates response [99]
Neutrophils/lymphocytes ratio	Cellular	Immune	58	Overall survival (NLR ≥ 4) HR = 2.2, *p* = 0.0009	Neutrophils and lymphocytes by flow cytometry High value indicates bad response [100]
Circulating Bim^+^PD-1^+^CD8^+^ T cells	Cellular	Immune	13	*p* < 0.05	Bim+PD-1+CD8+ T cell by flow cytometry High value indicates better response [101]
Total tumor infiltrating lymphocytes (TILs)	Cellular	Immune	64	*p* = 0.005	Total TILs by IHC High value indicates response [102,103]
Total eosinophils	Cellular	Immune	29	Progression-free survival *p* < 0.0001, overall survival p = 0.017	Absolute eosinophil counts by blood tests High values indicate better response [104]
Lactate Dehydrogenase (LDH)	Secreted	Serum	66	Overall survival *p* = 0.0292	LDH ELISA. Elevated value indicates bad response [105]
sCD25	Secreted	Serum	262	% survival, HR = 1.26, *p* < 0.0165	sCD25 level by sIL-2 Receptor EIA assay High value indicates bad response [106]
CXCL11	Secreted	Serum	247	Overall survival, HR = 1.88, *p* = 0.014	CXCL11 level examined by bead-based multiplexed immunoassay. High value indicates bad response [107]
CXCL9 and CXCL10	Secreted	Plasma	18	*p* < 0.001	CXCL9 and CXCL10 levels examined by ELISA. Levels after anti-PD1 + anti-CTLA4 treatment are higher in responders vs. non-responders [108]
C-reactive protein	Secreted	Serum	196	*p* = 0.028	CRP by immunofiltration High value indicates response [109]

**Table 4:** Examples of the relevancy of DNA methylation alterations as non-invasive diagnostic and prognostic biomarkers in cancer.

Type of Biomarker	Gene	Type of Cancer	Description	Accuracy of Panel Including Methylated Gene or *p* Value
Diagnostic	*ARF*	Bladder	Urine *ARF* promoter detects bladder cancer [192]	∆82%/96%
Prognostic	*APC, GSTP1*	Prostate	*APC* and *GSTP1* hypermethylation in prostate cancer strongly correlated to adverse pathological features [193]	ROC of the assay test score: clinical AUC = 0.79
Diagnostic	*BCL*	Bladder	Urine sediments *BCL* methylation detects bladder cancer [194]	† 78% (29/37)
Prognostic	*CDH13*	Prostate	Serum methylation of *CDH13* was significantly associated with advanced tumor stage, worse survival outcome and relative risk of death [195]	HR 6.132 (95%CI: 3.160–12.187) *p* = 0.0073
Diagnostic	*CDKN2A*	Bladder	Urine *CDKN2A* promoter detects bladder cancer [192]	∆82%/96%
Diagnostic	*DAPK*	Bladder	Urine sediments *DAPK* methylation detects bladder cancer [194]	† 78% (29/37)
Diagnostic (early)	*ERα*	Prostate/breast (primary)	Serum promoter *ERα* methylation detects early stage prostate and breast cancer [196,197]	∆75%/70%
Diagnostic (early)	*ERβ*	Prostate	Serum promoter *ERβ* methylation detects early stage prostate cancer [196]	∆75%/70%
Diagnostic	*FBN1*	Colorectal	Stool *FBN1* methylation detects colorectal cancer [198]	∆84.3%/93.3%
Diagnostic	*FBN2*	Colorectal (primary)	Serum methylation of *FBN2* detects colorectal cancer in males and hepatic metastasis [199]	Male: *p* = 0.0167; hepatic metastasis: *p* < 0.0001
Diagnostic, Prognostic	*GSTP1*	Bladder/prostate/castrate-resistant prostate/breast	Urine/serum *GSTP1* is hypermethylated in prostate cancer and strongly correlated to adverse pathological features [193,200,201]	∆82%,96%/−/† 82% (28/34)/∆75%/98%/† 6% 7/120/† 22% 22/101
Diagnostic	*FHIT*	Ductal breast cancer	Serum *FHIT* is associated with breast cancer [202]	*p* < 0.05
Diagnostic	*hMLH1*	Breast	Serum *hMLH1* detects breast cancer [203]	AUC = 0.727 (BCa versus NC), AUC = 0.789 (BCa versus BN)
Prognostic	*HLTF*	Colorectal	Serum *HLTF* methylation is associated with increased risk of recurrence [204]	HR 2.7 (95%CI: 1.2–6.0) *p*= 0.014)
Diagnostic	*HOXD13*	Breast	Serum *HOXD13* detects breast cancer [203]	AUC = 0.727 (BCa versus NC), AUC = 0.789 (BCa versus BN)
Diagnostic (early)	*5MCAM*	Prostate	Serum promoter *5MCAM* methylation detects early stage prostate cancer [196]	∆75%/70%
Diagnostic	*MGMT*	Bladder/lung/Colorectal	Clinical response to dacarbazine is restricted to those with *MGMT* hypermethylation in colorectal cancer [205]	∆82%/96%
Diagnostic	*NID2*	Bladder (primary)	Urine *NID2* methylation detects primary bladder cancer [206]	† 94% (466/496)
Diagnostic	*P16*	Breast	Serum *P16* detects breast cancer [203]	AUC = 0.727 (BCa versus NC), AUC = 0.789 (BCa versus BN)
Diagnostic	*PCDHGB7*	Breast	Serum *PCDHGB7* detects breast cancer [203]	AUC = 0.727 (BCa versus NC), AUC = 0.789 (BCa versus BN)
Prognostic	*PCDH10*	Prostate	*PCDH10* methylation in serum is an independent predictor of worse biochemical recurrence-free survival and overall survival [207]	HR 2.796 (95%CI: 1.431–6.763) *p* = 0.006
Diagnostic	*PCDH17*	Bladder	Urine sediment *PCDH17* methylation detects bladder cancer [208]	∆90%/93.96%
Diagnostic	*PHACTR3*	Colorectal	Stool *PHACTR3* methylation detects colorectal cancer [209]	Sensitivity: 55%–66%; specificity: 95%–100%
Diagnostic	*POU4F2*	Bladder	Urine sediment *POU4F2* methylation detects bladder cancer [208]	∆90%/93.96%
Diagnostic	*TERT*	Bladder	Urine sediments *TERT* methylation detects bladder cancer [194]	† 78% (29/37)
Diagnostic	*TMEFF2*	NSCLC	Higher frequency of *TMEFF2* methylation in tumors without *EGFR* mutations than those harboring *EGFR* mutations [210]	Multivariate adjusted odds ratio = 7.13 (95%CI: 2.05–24.83) *p* = 0.002
Diagnostic (early)	*RARB*	Prostate	Urine sediments *RARB* methylation detects early stage prostate cancer [211]	† 82% (28/34)
Diagnostic	*RARβ2*	Breast	Serum *RARβ2* promoter methylation as part of a methylation/specific PCR assay detects breast cancer [200]	† 6% 7/120/†22% 22/101
Diagnostic (early)	*RASSF1*	Prostate	Urine sediments *RASSF1* methylation detects early-stage prostate cancer [211]	† 82% (28/34)
Diagnostic, Prognostic	*RASSF1a*	Breast/lung/ovarian	Serum *RASSF1a* promoter methylation as part of a methylation/specific PCR assay detects breast cancer [200]	AUC = 0.727 (BCa versus NC), AUC = 0.789 (BCa versus BN)/† 6% 7/120/† 22% 22/101
Diagnostic, prognostic	*SEPT9, TAC, CEA*	Colorectal	Serum *SEPT9* methylation predicts colorectal cancer; Epipro Colon 2.0 with 2/3 algorithm is the most effective assay [212]. In postoperative serum, *SEPT9*, *CEA* or *TAC* methylation predict recurrence and survival [213]	(Diagnostic) Sensitivity = 0.71, Specificity = 0.92, AUC = 0.88. (Prognostic) Disease-free survival: adjusted hazard rations of the ∆ = 2.58–4.71 *p* < 0.05; recurrence: sensitivity = 32.6–90; specificity = 80–90
Diagnostic	*SFN*	Breast	Urine sediment *SFN* methylation detects bladder cancer [194]	AUC = 0.727 (BCa versus NC), AUC = 0.789 (BCa versus BN)
Diagnostic	*SNCA*	Colorectal	Stool *SNCA* methylation detects colorectal cancer [198]	∆84.3%/93.3%
Prognostic	*SST*	Colorectal	High serum *SST* methylation is an independent prognostic biomarker of colorectal cancer [214]	Multivariate adjusted for cancer-specific survival: HR 1.96 (95%CI: 1.06, 3.62) *p* = 0.031; for overall survival HR 2.60 (95%CI: 1.37, 4.94) *p* = 0.003
Diagnostic	*TWIST1*	Bladder (primary)	Urine *TWIST1* methylation detects primary bladder cancer [206]	† 94% (466/496)
Diagnostic, prognostic	*VIM*	Colorectal	Serum *VIM* methylation correlated with liver metastasis, peritoneal dissemination, and distant metastasis [215]	(Liver metastasis) *p* = 0.026 (Peritoneal dissemination) *p* = 0.0029 (Distant metastasis) *p* = 0.0063
Prognostic	*mir-34b/c*	Colorectal	Mucosal wash fluid *mir-34b/c* methylation is associated with invasiveness [216]	Accuracy: 91.3% for the training set and 85.1% for the test set.
Prognostic	*MGMT*	Glioblastoma multiforme	Serum and tumor methylation of *MGMT* is associated with better stable response [217]	Median time to progression: log-rank test, *p* = 0.006, 29.9 weeks with methylated *MGMT*, 95%CI, 24.3–35.4) vs. 15.7 weeks with unmethylated *MGMT* (95%CI, 14.3–17.2).
Diagnostic, Prognostic (early)	*Panel of 6 genes (CDO1, HOXA9, AJAP1, PTGDR, UNCX, and MARCH11)*	Lung	Methylation status in the 6 genes analyzed in serum for the detection of stage IA NSCLC. In addition, a prognostic risk category based on the cancer and serum methylation status of *CDO1*, *HOXA9*, *PTGDR*, and *AJAP1* refined the risk stratification for outcomes as an independent prognostic factor in early-stage disease [218]	(Serum) Sensitivity: 72.1%; specificity: 71.4%. (Prognosis factor) Combination methylation marker multivariate adjusted *p* = 0.035
Prognostic	*BRMS1*	Lung	Cell-free DNA circulating *BRMS1* promoter methylation has a statistically significant influence both on operable NSCLC patients’ disease-free interval (DFI) time and OS and on advanced NSCLC patients’ PFS and OS [219]	Multivariate analysis: for progression-free survival: HR 1.951 (95%CI: 1.175–3.238) *p* = 0.01; for overall survival: HR 2.057 (95%CI: 1.247–3.386) *p* = 0.005
Prognostic	*SOX17*	Lung	SOX17 promoter methylation in plasma cell-free DNA has a statistically significant influence on advanced NSCLC patient overall survival [220]	Univariate analysis for overall survival: HR 1.834 (95%CI: 1.105–3.045) *p* = 0.019

† Overall detection level.
